# Left Hepatic Artery Pseudoaneurysm and Arteriobiliary Fistula in an Adolescent: A Case Report

**DOI:** 10.7759/cureus.108463

**Published:** 2026-05-07

**Authors:** Aamir Ghazanfar, Syeda Ambreen Gulab, Wajiha Arshad, Ania Javed, Mahjabeen Mehmood

**Affiliations:** 1 General Surgery, Kahuta Research Laboratories (KRL) Hospital, Islamabad, PAK; 2 Surgery, Kahuta Research Laboratories (KRL) Hospital, Islamabad, PAK; 3 Diagnostic Radiology, Kahuta Research Laboratories (KRL) Hospital, Islamabad, PAK; 4 Radiology, District Headquarters (DHQ) Hospital, Haripur, PAK; 5 Radiology, Kahuta Research Laboratories (KRL) Hospital, Islamabad, PAK

**Keywords:** angioembolization, arterio-biliary fistula, arteriovenous malformation, hemangioma, left hepatic artery pseudoaneurysm

## Abstract

Hepatic artery pseudoaneurysm is a rare but potentially life-threatening vascular condition, with risks of rupture, fistula formation, and massive hemorrhage. It most commonly occurs secondary to trauma, infection, inflammation, or iatrogenic injury.

In this report, the case of a 15-year-old boy with idiopathic juvenile arthritis who presented with chronic anemia, intermittent right upper quadrant and epigastric pain, low-grade fever, and occasional vomiting for two years is reported, with repetitive attacks of pancreatitis. Ultrasound of the abdomen showed sludge in the gallbladder. Magnetic resonance cholangiopancreatography (MRCP) showed the lumen of the gallbladder with an ill-defined fluid level suggestive of a sludge ball, and a wall thickness of 3 mm. So, a laparoscopic cholecystectomy was performed on the basis of the patient’s symptoms and findings. Postoperatively, after one month, the patient had symptoms of abdominal pain, melena, and a fall in hemoglobin. CT scan and selective angiography revealed a small pseudoaneurysm arising from the medial branch of the left hepatic artery with associated biliary duct dilatation, suggestive of an arteriobiliary fistula. The patient underwent transcatheter coil embolization. Post-embolization angiography confirmed complete occlusion of the pseudoaneurysm with preserved hepatic arterial flow.

Early recognition and timely endovascular intervention are essential to prevent life-threatening complications, including rupture and massive hemorrhage.

## Introduction

A pseudoaneurysm results from the disruption of the arterial wall, leading to blood leakage outside the vessel lumen that is contained by surrounding tissues rather than the normal arterial wall layers. Unlike a true aneurysm, a pseudoaneurysm does not involve all layers of the vessel wall [1].

Hepatic artery pseudoaneurysms are rare but clinically significant due to their high risk of rupture and hemorrhage. They most commonly occur secondary to trauma, infection, inflammation, pancreatitis, or iatrogenic vascular injury during hepatobiliary procedures [2,3]. Among visceral artery pseudoaneurysms, the splenic artery is most frequently involved, whereas involvement of the left hepatic artery is particularly rare [4,5]. Early diagnosis is essential, as untreated pseudoaneurysms may result in catastrophic bleeding.

Advances in imaging modalities, particularly computed tomography (CT) angiography and selective angiography, have improved the diagnosis and management of these lesions [6]. A pseudoaneurysm occurs due to disruption of arterial wall integrity, resulting in blood collection outside the vessel lumen that is contained by surrounding tissues [1]. Most hepatic artery pseudoaneurysms arise secondary to trauma (iatrogenic or blunt), infection or inflammation, and acute or chronic pancreatitis [7,8]. Less common causes include vascular disorders such as fibromuscular dysplasia and mycotic infection [9].

In children, hepatic artery pseudoaneurysms are rare and differ in etiology compared to adults. They more commonly arise from inflammatory injury rather than atherosclerotic or degenerative disease. Contributing factors may include congenital or structural vascular fragility (less common unless syndromic features are present) and systemic inflammatory or vasculitic disorders. Unlike adults, pediatric cases are less frequently associated with trauma or prior procedures and are more likely to have an immune-mediated or inflammatory basis, particularly in the setting of pancreatitis.

Recurrent acute pancreatitis plays an important role in the pathogenesis, where peripancreatic inflammation can lead to enzymatic erosion and weakening of the arterial wall, ultimately resulting in pseudoaneurysm formation. However, in pediatric patients, recurrent pancreatitis itself often has an identifiable underlying cause, such as genetic mutations (e.g., PRSS1, SPINK1, CFTR), structural anomalies, or autoimmune pancreatitis, particularly when associated with elevated IgG4 levels. This suggests that vascular complications may be secondary to an underlying systemic or inflammatory disorder rather than isolated pancreatic disease. Additionally, systemic inflammatory conditions such as IgG4-related disease and juvenile idiopathic arthritis have been implicated, with rare reports of hepatic artery involvement.

In a review of 27 hepatic artery pseudoaneurysms, left hepatic artery involvement was reported in approximately three cases (11.1%) [10]. These are considerably less frequent than pseudoaneurysms of the right hepatic or common hepatic artery.

## Case presentation

A 15-year-old boy with a history of idiopathic juvenile arthritis presented with chronic anemia, intermittent epigastric and right upper quadrant pain, occasional vomiting, and intermittent low-grade fever. His symptoms had worsened over the past month.

There were no associated symptoms of bleeding, bruising, or skin discoloration. He had a history of multiple hospitalizations since birth for pancreatitis, as well as lymphadenitis, bilateral cervical lymphadenopathy, and splenomegaly. There was no history of birth trauma, fractures, therapeutic or diagnostic venipuncture, or intra-abdominal cannulation. He had not undergone any surgical procedures.

On physical examination, his height and weight were appropriate for his age. He appeared pale and had bilateral enlarged cervical lymph nodes. The right upper quadrant and epigastric regions were tender. The remainder of the systemic examination was unremarkable.

Investigations

Complete blood count revealed a hemoglobin level of 6.7 g/dL (post-transfusion: 8.4 g/dL), with normal platelet and white blood cell counts. Erythrocyte sedimentation rate, coagulation profile, C-reactive protein, renal function tests, liver function tests, lipid profile, and protein C and S levels were within normal limits. Serum amylase and lipase levels were elevated, along with a recent increase in immunoglobulin G4 levels. Echocardiography was unremarkable (Table 1).

**Table 1:** Investigations

Category	Parameter/Test	Result	Unit	Reference Range/Remarks
Complete blood count	Total leukocyte count	10,400	/µL	-
	Hemoglobin	6.7/8.4 (post-transfusion)	g/dL	-
	Platelets	180,000	/µL	-
Liver function tests (LFT)	Bilirubin	1.36	mg/dL	-
	Alanine aminotransferase	86	U/L	-
	Aspartate aminotransferase	124	U/L	-
	Serum amylase	684	U/L	-
	Serum lipase	410	U/L	-
	IgG4	3279.9	mg/L	37-1360 mg/L (12-14 years)

Abdominal ultrasonography showed sludge in the gallbladder. Contrast-enhanced CT of the abdomen and pelvis demonstrated a bulky pancreas without any focal enhancing lesion in the arterial, venous, or delayed phases. The pancreatic duct was not dilated, and no pancreatic cyst, mass, or growth was identified. There was no evidence of vascular thrombosis, ascites, or pleural effusion. A small, well-defined, rounded lesion in segment III of the liver, appearing isodense to hypodense, showed intense homogeneous enhancement in the arterial phase with contrast retention, suggestive of hepatic hemangioma. Splenomegaly with multiple ill-defined hypodense splenic lesions and mild abdominopelvic lymphadenopathy were noted, raising suspicion for a lymphoproliferative disorder.

Magnetic resonance cholangiopancreatography (MRCP) demonstrated an ill-defined fluid level within the gallbladder lumen, suggestive of a sludge ball, with a wall thickness of 3 mm. Subtle pancreatic prominence and tortuosity of intrahepatic ducts in the left lobe were also observed, with findings suggestive of hemangioma.

**Figure 1:**
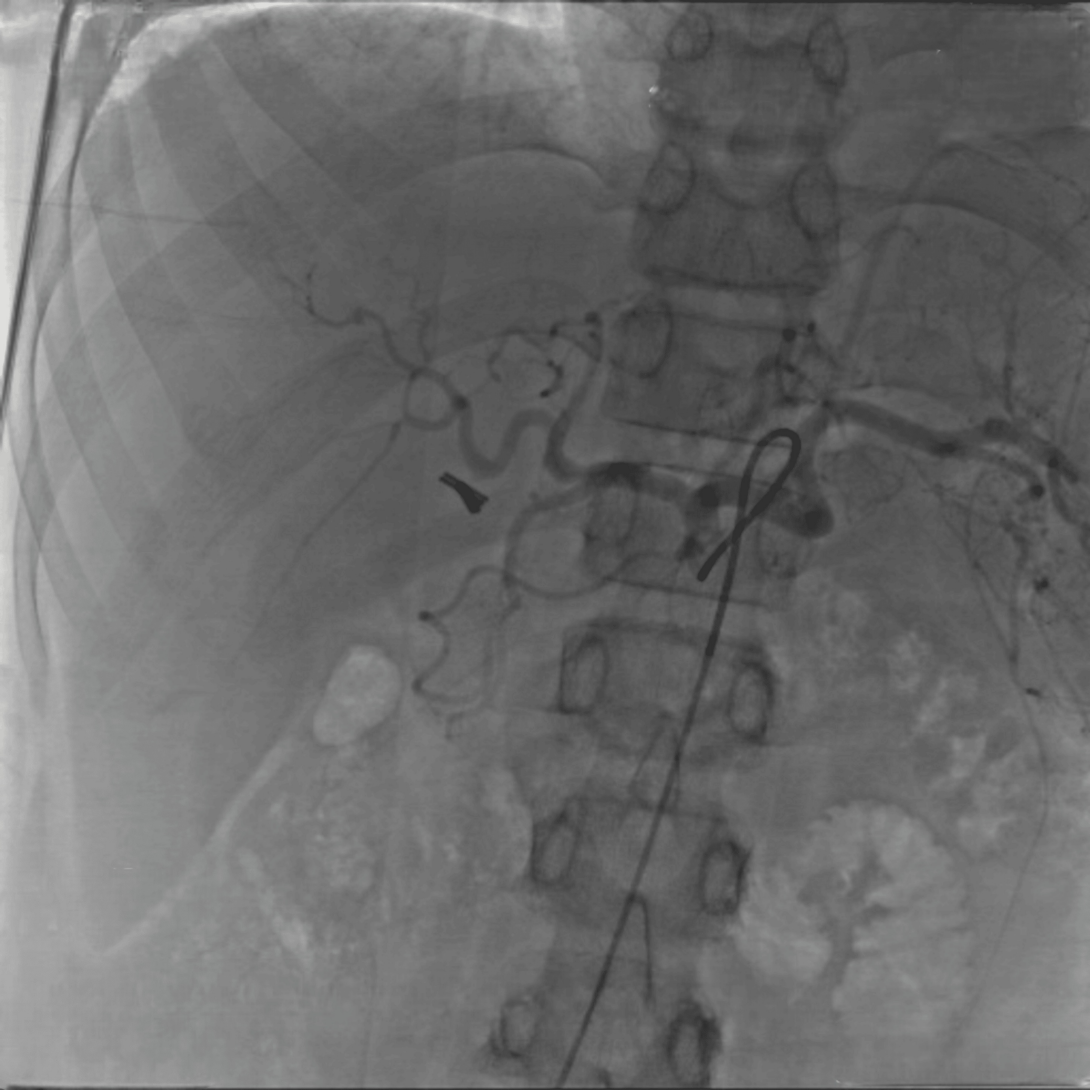
Angiogram taken from left hepatic artery

**Figure 2:**
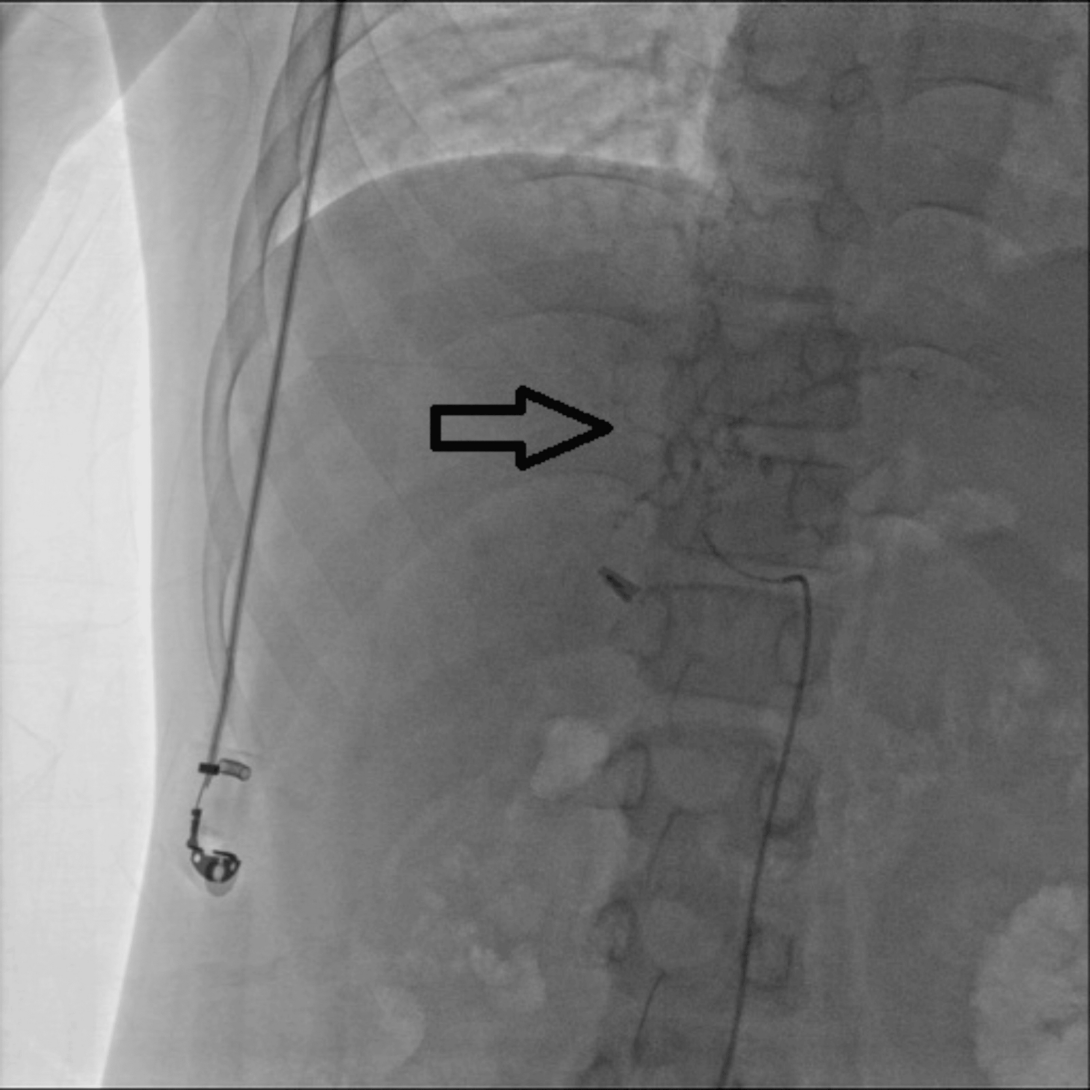
Selective angiographic run of the left hepatic artery demonstrating a small aneurysm arising from its medial branch (arrow)

**Figure 3:**
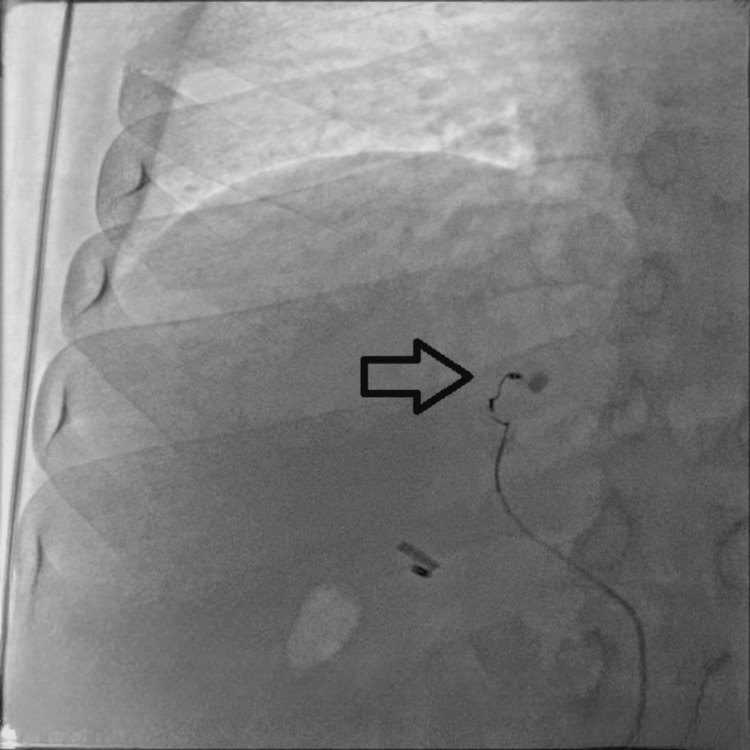
Micro coil deployed in feeding artery with stasis of contrast in pseudoaneurysm (arrow)

Selective angiography was performed from the left hepatic artery (Figure 1). A subsequent selective run of the left hepatic artery demonstrated a small aneurysm arising from its medial branch (Figure 2). Further angiographic imaging confirmed these findings (Figure 3).

Differential diagnosis

The differential diagnosis for hepatic artery pseudoaneurysm includes hepatic hemangioma, arteriovenous malformations, and true hepatic artery aneurysm.

Treatment

Left hepatic artery pseudoaneurysm is a rare but potentially life-threatening condition due to the risk of rupture and hemorrhage. Management depends on symptoms, lesion size, etiology, and patient stability; however, treatment is indicated even in asymptomatic cases [11]. Transcatheter coil embolization is the preferred first-line treatment in most cases [1,6].

Early laparoscopic cholecystectomy was performed in the current case to prevent complications. Intraoperative findings included an overdistended gallbladder containing sludge and blood clots. The patient remained stable throughout hospitalization and was discharged on home medications and scheduled follow-up.

A CT scan performed one month later demonstrated a left hepatic artery pseudoaneurysm, for which coil embolization was undertaken. A 4 Fr sheath was placed in the right common femoral artery. A 4 Fr C2 catheter and Terumo Glidewire (Terumo Corporation, Tokyo, Japan) were used to cannulate the celiac axis, and angiographic runs were obtained. A round contrast-filled structure was identified arising from the medial branch of the left hepatic artery. A 2.7 Fr microcatheter was used to access the feeding vessel. After confirming the catheter position, two coils (2-3 mm) were deployed, resulting in stasis of contrast within the pseudoaneurysm. Subsequent angiography demonstrated preserved perfusion of the right lobe, the lateral branch of the left hepatic artery, and the gastroduodenal artery. The catheter and sheath were removed, and hemostasis was achieved with manual compression. No complications were observed.

**Figure 4:**
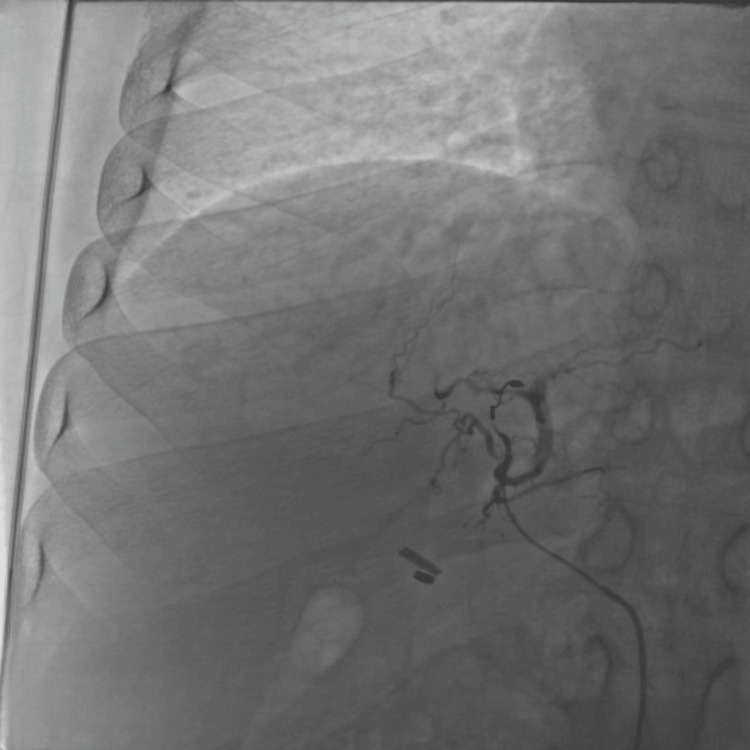
Selective run taken from the left hepatic artery showing blockage of feeding artery

Angiographic image shows coil embolization of the feeding artery of the pseudoaneurysm (Figure 4).

Outcome and follow-up

The patient remained hospitalized for two days for a laparoscopic cholecystectomy and was discharged on postoperative day 1. He was prescribed antibiotics for five days. The patient presented 10 days post-procedure for stitch removal. At the second follow-up, two weeks later, he developed symptoms suggestive of a left hepatic artery pseudoaneurysm with an arterio-biliary fistula, which was confirmed on CT angiography. CT angiography performed one month after surgery demonstrated findings suggestive of a left hepatic artery pseudoaneurysm with an arteriobiliary fistula.

In the present case, although the left hepatic artery pseudoaneurysm was small, early coil embolization was performed to prevent rupture and thromboembolic complications [6,11]. The postoperative course was uneventful, and the patient was discharged with scheduled follow-up. On the fourth day following coil embolization, color Doppler imaging demonstrated an endogenous round lesion with a surrounding hypoechoic area and absence of color flow, consistent with a thrombosed pseudoaneurysm.

The patient was reassured and continued follow-up with Doppler ultrasound and serial hemoglobin measurements. Hemoglobin levels remained stable during subsequent evaluations. A thorough diagnostic workup is essential to accurately differentiate between true and false aneurysms.

## Discussion

A pseudoaneurysm is a contained arterial rupture in which blood escapes through the vessel wall and is confined by surrounding tissues, unlike a true aneurysm that involves all layers of the vessel wall [1]. Left hepatic artery pseudoaneurysm is rare but potentially life-threatening due to the risk of rupture and hemorrhage [3,4]. Most cases are secondary to iatrogenic injury, trauma, infection, or arteriovenous malformations [2,8,9]. Disruption of the arterial wall leads to blood extravasation, forming a pulsatile hematoma that communicates with the parent artery [1]. Continuous arterial pressure predisposes the pseudoaneurysm to rapid enlargement and rupture.

Clinical presentation may be variable or delayed, including right upper quadrant or epigastric pain and gastrointestinal bleeding (hematemesis or melena). Quincke’s triad - abdominal pain, jaundice, and upper gastrointestinal bleeding - is characteristic but uncommon [6]. In severe cases, rupture may lead to hypotension.

Regarding etiology in this case, recurrent acute pancreatitis may be a contributing factor. The mechanism involves peripancreatic inflammation, enzymatic erosion, weakening of the arterial wall, and pseudoaneurysm formation. IgG4-related disease and juvenile arthritis may also be contributing factors; however, hepatic artery involvement, though rare, has been reported.

CT angiography is the investigation of choice and typically demonstrates a contrast-filled sac with arterial communication [6]. Transcatheter coil embolization is the treatment of choice, as it is minimally invasive, has a high success rate, and preserves hepatic perfusion when feasible [1,11]. If endovascular therapy fails, surgical management, including ligation of the left hepatic artery or aneurysm excision, is indicated [10].

Complications include rupture with massive intra-abdominal or gastrointestinal bleeding, hepatic ischemia or infarction, infection or abscess formation, and rebleeding [3]. Prognosis is favorable with early diagnosis and prompt treatment.

## Conclusions

Hepatic artery pseudoaneurysms are exceedingly rare but potentially life-threatening due to the risk of rupture, fistula formation, and massive hemorrhage. Prompt diagnosis with contrast-enhanced CT angiography is essential to prevent complications. Early intervention with coil embolization is an effective modality with a high success rate.

In this case, successful coil embolization resulted in complete thrombosis of the pseudoaneurysm with preservation of hepatic arterial flow and an uneventful recovery. Early endovascular intervention remains a safe and effective strategy for preventing catastrophic hemorrhagic complications.
